# Effects of diclofenac, sulfamethoxazole, and wastewater from constructed wetlands on *Eisenia fetida*: impacts on mortality, fertility, and oxidative stress

**DOI:** 10.1007/s10646-023-02690-3

**Published:** 2023-08-26

**Authors:** Justyna Drzymała, Joanna Kalka

**Affiliations:** 1 The Biotechnology Centre, Silesian University of Technology, Gliwice, Poland; 2 Environmental Biotechnology Department, Faculty of Energy and Environmental Engineering, Silesian University of Technology, Gliwice, Poland

**Keywords:** Diclofenac, Earthworm, Mortality, Reproduction, Sulfamethoxazole, Wastewater

## Abstract

Soil contamination with micropollutants is an important global problem and the impact of these pollutants on living organisms cannot be underestimated. The effects of diclofenac (DCF) and sulfamethoxazole (SMX), their mixture (MIX), and wastewater containing these drugs on the mortality and reproduction of *Eisenia fetida* were investigated. The impact on the activities of antioxidant enzymes in earthworm cells was also assessed. Furthermore, the influence of the following parameters of the vertical flow constructed wetlands on wastewater toxicity was investigated: the dosing system, the presence of pharmaceuticals and the plants *Miscanthus giganteus*. The compounds and their mixture significantly affected the reproduction and mortality of earthworms. The calculated values of LC_50,28 days_ values were 3.4 ± 0.3 mg kg^−1^ for DCF, 1.6 ± 0.3 mg kg^−1^ for SMX, and 0.9 ± 0.1 mg kg^−1^ for MIX. The EC_50_ (reproduction assay) for DCF was 1.2 ± 0.2 mg kg^−1^, whereas for SMX, it was 0.4 ± 0.1 mg kg^−1^, and for MIX, it was 0.3 ± 0.1 mg kg^−1^, respectively. The mixture toxicity index (MTI) was calculated to determine drug interactions. For both *E. fetida* mortality (MTI = 3.29) and reproduction (MTI = 3.41), the index was greater than 1, suggesting a synergistic effect of the mixture. We also observed a negative effect of wastewater (raw and treated) on mortality (32% for raw and 8% for treated wastewater) and fertility (66% and 39%, respectively) of *E. fetida*. It is extremely important to analyze the harmfulness of microcontaminants to organisms inhabiting natural environments, especially in the case of wastewater for irrigation of agricultural fields.

## Introduction

Drugs and their metabolites have been detected in the natural environment for many years. Their presence has been confirmed not only in surface water and soil, but also in groundwater, drinking water, and sediment. Pharmaceuticals (PhCs) reach the natural environment mainly through treated sewage or sediments from wastewater sludge (Bisognin et al. 2021). Painkillers and anti-inflammatory drugs are the most commonly detected pharmaceuticals and personal care products (PPCPs) in the environment due to their high consumption and over-the-counter availability (Yang et al. 2017a). The second group of drugs, frequently used in human and veterinary medicine, is sulfonamide antibiotics, which are mostly used in bacterial infections. Pharmaceutical residues can easily reach the soil environment, e.g., through the release of animal manure as fertilizer (Gros et al. 2019; Pan and Chu 2017). However, the main source of PPCPs in soil is wastewater treatment plant. Most sewage sludges are released onto agricultural land, where they are used as biosolids to increase soil nutrient levels and agricultural yields. In addition, treated wastewater is also used for soil irrigation (Zhang et al. 2021).

This work focuses on the impacts of two pharmaceuticals on the earthworm *Eisenia fetida*. Diclofenac (DCF) is a commonly used non-steroidal anti-inflammatory drug (NSAID) and is applied as a pain reliever. It is administered mainly orally in tablets or applied to the skin in the form of an ointment. In the case of an oral dose, 65–70% is excreted in the urine and 20–30% in the feces as the parent drug or as metabolites (Lonappan et al. 2016). In the European Union, DCF has been classified as a priority substance and is included in the substance watch list (EC 2015; WFD 2000). Sulfamethoxazole (SMX) is a representative of sulfonamide antibiotics, which are used against a wide spectrum of bacterial infections. As antibiotics are not completely metabolized in humans and animals, high percentages are released into water and soil (Cycoń et al. 2019). The maximum concentration of SMX in single-house wastewater can be as high as 4.4 mg L^−1^ (Abegglen et al. 2009). The presence of SMX in the natural environment can result in antibiotic resistance of bacteria, which poses a risk to the natural environment (Felis et al. 2020). Table 1 shows the environmental concentrations of DCF and SMX.

**Table 1:** The maximum environmental concentration detected for DCF and SMX

Compound	Environment	Concentration	Country	References
DCF	Surface water	57.16 µg L^−1^	Nigeria	Sathishkumar et al. (2020)
	Groundwater	13.48 µg L^−1^	Nigeria	Sathishkumar et al. (2020)
	Sea water	843 ng L^−1^	China	Bonnefille et al. (2018)
	Soil	257 µg kg^−1^	Pakistan	Ashfaq et al. (2019)
	Wastewater—influent	22.3 µg L^−1^	South Africa	Agunnbiade and Moodley (2016)
	Wastewater—effluent	19.0 µg L^−1^	South Africa	Agunnbiade and Moodley (2016)
SMX	Surface water	22 µg L^−1^	Kenya	Straub (2016)
	Groundwater	1.11 µg L^−1^	Denmark	Lapworth et al. (2012)
	Sea water	48 ng L^−1^	United Kingdom	Baran et al. (2011)
	Soil	54.5 µg kg^−1^	China	Cycoń et al. (2019)
	Wastewater—influent	1340 µg L^−1^	Taiwan	Baran et al. (2011)
	Wastewater—effluent	6.0 µg L^−1^	United States	Baran et al. (2011)

Conventional wastewater treatment plants are designed to improve the hygienic conditions of the water and remove degradable organic compounds and nutrients such as nitrogen and phosphorus compounds. The use of various chemicals (pharmaceuticals, pesticides, corrosion inhibitors, and heavy metals) creates new challenges for conventional treatment systems. Wastewater treatment plants based on activated sludge, membrane bioreactors, and separation membranes require expensive technologies that are often not used in rural areas (Wu et al. 2015). Systems that use coagulation, flocculation, adsorption on activated carbon, ozonation, or advanced oxidation processes are characterized by high investment and operating costs (Luo et al. 2014). For these reasons, inexpensive and effective methods of removing microcontaminants from the environment are constantly being searched for.

Constructed wetlands (CW) are artificially constructed wetlands that combine biological, chemical, and physical processes that occur under natural conditions (Vymazal et al. 2021). The natural receiver of wastewater treated in wetland systems is the soil. Given that the design of such systems is in line with current policies of sustainable development, treated water and nutrients contained in wastewater can be safely used to improve soil quality. However, to safely use wastewater for irrigation, it must be ensured that it contains no micropollutants.

The pharmaceuticals mentioned above are not completely mineralized in CW, but are transformed to intermediate products through various metabolic pathways (Sochacki et al. 2018). In a previous study, the efficiency of DCF removal in CW ranged from 0 to 96% (Gorito et al. 2017), while other studies have reported SMX removal efficiencies of 15–91% (Dan et al. 2013; Sochacki et al. 2018). However, from an environmental point of view, the most significant parameter is not a removal efficiency but wastewater detoxification (reduction of toxicity), making it important to verify the negative impacts of various substances and wastewaters on model organisms.

In this study, we investigate the impacts of selected pharmaceuticals on the earthworm *Eisenia fetida*. Earthworms are in close contact with soil particles through their permeable skin and lipophilic intestines and are therefore highly affected by pollutants reaching the soil system. Earthworms are important soil invertebrates that participate in nutrient cycling and in the formation of the soil profile. Because they are in permanent contact with soil particles, earthworms are highly suitable as monitoring organisms (Bartlett et al. 2010). *Eisenia fetida* earthworms, although they are organisms that mainly decompose organic waste, are very often used as representatives of the soil environment and used to present the environmental risk resulting from the presence of various pollutants in the soil (Li et al. 2020b; Lin et al. 2014). In this study, we determined the effects of DCF and SMX on *Eisenia fetida* mortality and reproduction and investigated the antioxidant enzyme activity in earthworm cells when exposed to these compounds. In the first stage of the investigation, pharmaceutical solutions and their mixtures were used. In the second stage, wastewater from vertical flow CWs (containing DCF and SMX) was analyzed. In addition, the influences of the technological parameters of the CW on wastewater toxicity were investigated: the dosing system, the presence of pharmaceuticals, and the presence of the plant species *Miscanthus giganteus*.

## Materials and methods

### Tested pharmaceuticals: diclofenac and sulfamethoxazole

We selected two commonly detected pharmaceuticals for the study: diclofenac (DCF, CAS number: 15307-86-5) and sulfamethoxazole (SMX, CAS: 723-46-6). Both compounds were purchased from Sigma-Aldrich (purity above 99%). Additionally, we analyzed a binary mixture (MIX) of these compounds. The basic properties of the tested substances are summarized in Supplementary Table SI.1.

### Constructed wetlands

Vertical flow constructed wetlands (CW) as experimental systems have been described in detail in our previous research by Drzymała et al. (2022) and Sochacki et al. (2018). Briefly, the laboratory model consisted of 24 columns (filled with gravel, quartz sand and a mixture of sand and organic soil; Supplementary Fig. SI.1).

Synthetic municipal wastewater (control), prepared according to the modified Nopens et al. (2001) protocol, was administered to each experimental column. The tested wastewater (artificially spiked) also contained a mixture of DCF and SMX (2 mg L^−1^ of each).

The selected concentrations of pharmaceuticals (2 mg L^−1^ of each) were based on our previous study (Sochacki et al. 2018). The results of preliminary studies were promising (studies were carried out at lower concentrations: 0.5 mg L^−1^ for DCF and SMX, respectively) taking into account the efficiency of the removal of pharmaceutical compounds; therefore, it was decided to increase the concentration of contaminants (from 0.5 to 2 mg L^−1^) in the following research. Higher concentrations of the pharmaceuticals analyzed may occur in point sources of pollution, which are undoubtedly sewage from single-family houses or hospitals (Felis et al. 2020; Yang et al. 2017a). We also wanted to stimulate the maximum negative effect (worst case scenario) that can be observed, for example, during the failure of transmission or sewage treatment systems.

We examine the impacts of the following three technological parameters on wastewater toxicity: the frequency of wastewater dosing (sewage dispersed twice a week in a 2.5 L volume and with a hydraulic loading rate HLR = 80 L d^−1^m^−2^ or five times a week in a volume of 1 L, HLR = 32 L d^−1^m^−2^), the presence of DCF and SMX (2 mg L^−1^ of each) in wastewater, and the presence of the *Miscanthus giganteus* plant species. Eight wastewater sample sets were collected and analyzed in triplicate. The description of the columns used in the experiment is presented in Table 2. The pollutant removal efficiencies are summarized in Supplementary Table SI.2.

**Table 2:** Description of columns used in the experiment

Columns description	Frequency of wastewater dosing	Presence of PhCs	Presence of *M. giganteus*
*Rack 1*			
R1-CTRL-Planted	2 times a week in a volume of 2.5 L, TWV = 5.0 L, HLR = 80 L d^−1^ m^−2^	–	+
R1-PhC-Planted		+	
R1-CTRL-Unplanted		–	–
R1-PhC-Unplanted		+	
*Rack 2*			
R2-CTRL-Planted	5 times a week in a volume of 1.0 L, TWV = 5.0 L, HLR = 32 L d^−1^ m^−2^	–	+
R2-PhC-Planted		+	
R2-CTRL-Unplanted		–	–
R2-PhC-Unplanted		+	

*TWV* total weekly volume of wastewater, *HLR* hydraulic loading rate, *R1* rack 1, *R2* rack 2, *CTRL* control wastewater (without DCF and SMX), *PhC* wastewater enriched with DCF and SMX, *Planted* columns with *Miscanthus giganteus*, *Unplanted* columns without *Miscanthus giganteus*

### Mortality and reproduction test for *E. fetida*

Studies on the influences of tested pharmaceuticals and wastewater on *E. fetida* mortality and reproduction were carried out according to OECD (2016). An increase in the mortality of adult earthworms (after 28 days of incubation) or a decrease in the number of offspring (after 56 days of incubation) compared to the control conditions indicate a negative effect of the tested solutions on *E. fetida*.

Earthworm mortality and reproduction tests were performed using organisms from own breeding. The organisms were purchased from a registered culture of *E. fetida* (Kępa Oborska, Poland, vet no. aPL14188401) and stored in a 50-L container at a temperature of 20 ± 2 °C and a light/dark cycle of 16 h/8 h.

The reference soil was prepared according to OECD (2016). The composition of the artificial soil is presented in Supplementary Table SI.3. The 500 g of artificial soil (pH = 5.8 ± 0.3) was placed in the test container and subsequently the test solution was added at appropriate concentrations, maintaining a 50% water holding capacity (WHC). Only organisms that were able to reproduce (with a visible *clitellum*) were selected for the experiments. The *E. fetida* test was performed in three replicates. For each repetition, 10 adult earthworms (weight between 300 and 600 mg) were used.

Concentrations causing 50% of the mortality of *E. fetida* (LC_50_, lethal concentration) and inhibiting reproduction (EC_50_, effect concentration) were calculated using the logarithmic probit analysis method and regression analysis using the Matlab^®^ 2013 software (The MathWorks, Inc.).

Preliminary tests were performed using solutions of the selected pharmaceuticals (DCF, SMX, and MIX) and the main experiment using wastewater from CW (influent and effluent). In the case of pharmaceutical solutions, the artificial soil was contaminated with an appropriate concentrations range of 0.008–2 mg kg^−1^ for DCF and SMX and 0.016–4 mg kg^−1^ for MIX (watered with distilled water to achieve 50% WHC). The MIX concentration means a binary mixture of DCF and SMX (e.g. a concentration of 4 mg kg^−1^ of MIX means a concentration of DCF of 2 mg kg^−1^ and SMX of 2 mg kg^−1^ - giving a total concentration of 4 mg kg^−1^ of MIX).

The artificial soil was watered with distilled water twice a week to maintain constant soil moisture (50% WHC). The proposed concentration ranges corresponded to the concentrations of these pharmaceuticals detected in the environment (Table 1) and with the concentrations of PhCs used in the CW experiment.

In the experiment with synthetic wastewater from CWs, both raw and treated sewage was analyzed. Artificial soil was watered with sewage at the beginning of the experiment and then twice a week to maintain constant soil moisture (50% WHC). The initial doses of pharmaceuticals in the samples watered with raw wastewater were as follows: on average, 0.31 mg kg^−1^ for DCF and 0.43 mg kg^−1^ for SMX; when treated sewage was used for watering, the doses were 0.12 mg kg^−1^ for DCF (range 0.01–0.27 mg kg^−1^) and 0.05 mg kg^−1^ for SMX (range 0.00–0.18 mg kg^−1^), depending on the removal efficiency of PhC removal in CWs. To simulate the conditions that may occur when sewage is used for irrigation of agricultural fields, the artificial soil was watered twice a week with analyzed sewage samples in a volume appropriate to obtain 50% WHC. Consequently, the doses of the pharmaceuticals in the soil increased throughout the experiment. After the first part of the toxicity study (mortality study – 28 days), the doses obtained in the soil were the following: for raw wastewater, 0.86 mg kg^−1^ for DCF and 1.20 mg kg^−1^ for SMX, and for treated wastewater, 0.30 mg kg^−1^ for DCF (0.03–0.78 mg kg^−1^) and 0.13 mg kg^−1^ for SMX (0.01–0.51 mg kg^−1^). After the second part of the investigation (reproduction study – 56 days), the doses obtained in the soil were the following: for raw wastewater, 1.42 mg kg^−1^ for DCF and 1.97 mg kg^−1^ for SMX, and for treated wastewater, 0.52 mg kg^−1^ for DCF (0.04–1.26 mg kg^−1^) and 0.21 mg kg^−1^ for SMX (0.02–0.84 mg kg^−1^).

Chicken manure (CDN, Poland) was used as food for earthworms throughout the experiment in an amount of 5 g/test container, added on the first day of the experiment and refilled once a week. The earthworms were incubated for 56 days. After 28 days of incubation, the adult organisms were removed, counted, and weighed. The containers containing *E. fetida* cocoons were incubated for another 28 days under the same experimental conditions. After this period, the number of juvenile *E. fetida* was determined.

### Activities of the antioxidant enzymes catalase and superoxide dismutase

Adult *E. fetida* removed from the test containers after 28 days of incubation were used to determine the activities of antioxidant enzymes. The three selected organisms of each test container (without external tissue damage) were used to prepare enzyme homogenates in a Pro200 homogenizer (Pro Scientific Inc. USA). Subsequently, homogenates were centrifuged (10 min, 10,000 rpm, 4 °C) to obtain supernatants for enzymatic analyzes. Appropriate buffers were used to prepare the supernatants to determine the activity of specific enzymes.

An Evolution 220 spectrophotometer (Thermo Fisher Scientific, Poland) was used to determine catalase (CAT, EC 1.11.1.6) and superoxide dismutase (SOD, E.C. 1.15.1.1) as well as protein concentrations using the Bradford method (Bradford 1976). The CAT activity was measured by the Góth method (Góth 1991) with 0.06 M sodium phosphate buffer (pH 7.4). This method is based on the decomposition of hydrogen peroxide by catalase in 1 min. The amount of remaining H_2_O_2_ can be measured spectrophotometrically at 405 nm. The SOD activity was determined using 0.05 M carbonate buffer (pH 10.2) (Misra and Fridovich 1972), based on the oxidation of adrenaline in 4 min, and the final activity was measured spectrophotometrically at 480 nm.

### Calculation of the mixture toxicity index value

The interactions between the components of the mixture were determined on the basis of the mixture toxicity index (MTI) value. All calculations were made using the following equations (Nagata et al. 2008):

1 $$M = {\sum} \,{f\left( i \right)} = {\sum} {\frac{{C\left( i \right)}}{{EC_{50}\left( i \right)}}}$$

2 $$M_0 = \frac{M}{{f_{{\rm{MAX}}}}}$$

3 $$MTI = 1 - \left( {\frac{{\log M}}{{\log M_0}}} \right)$$

where *C*(i) is the concentration of the *i*th component of the mixture; *EC*_50_(i) concentration of the *i*th component that has a 50% effect on model organisms; *f*_MAX_ the largest value of *f*(*i*) in the mixture.

Based on the above equations, it is possible to calculate the MTI value. Possible interactions in the mixture of substances are classified in Supplementary Table SI.4.

### Statistical analysis

All statistical tests were performed using STATISTICA 13 software (StatSoft Inc., 2016). Statistical analyzes were first performed using the Shapiro-Wilk test to verify whether the variables examined have a normal distribution. Second, one of two tests was selected: Student’s *t*-test (*α* = 0.05) or Mann–Whitney *U* test (*p* < 0.05). Independent groups of data were compared using Mann–Whitney *U* because the datasets analyzed were mostly nonnormally distributed. A Student’s *t*-test was used to compare groups that had a normal distribution.

## Results

### Mortality and reproduction of *E. fetida*

**Fig. 1:**
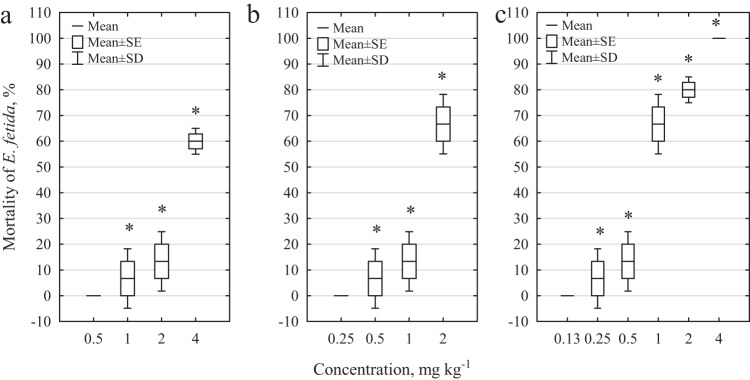
Mortality of *E. fetida*. **a** effect of DCF, **b** effect of SMX, and **c** effect of DCF and SMX mixture. * statistically significant differences compared to the control sample; Mann–Whitney *U* test, *p* < 0.05

The effects of the pharmaceuticals tested on earthworm mortality after 28 days of incubation are presented in Fig. 1 and in Supplementary Table SI.5. Toxic effects were observed only at the highest concentrations; at lower concentrations (for DCF, 0.008–0.5 mg kg^−1^, for SMX, 0.008–0.25 mg kg^−1^, and for MIX, 0.016–0.125 mg kg^−1^), no effect on *E. fetida* mortality was observed. For DCF, earthworm survival after 28 days with the dose of 2 mg kg^−1^ was 86.7%; for SMX, it was only 33.3%, and for MIX at 4 and 2 mg kg^−1^, survival was 0.0% and 20.0%, respectively. To determine the LC_50_ value for DCF, an additional experiment was performed, using a concentration of 4 mg kg^−1^. The calculated LC_50,28 days_ for DCF was 3.4 ± 0.3 mg kg^−1^; for SMX, it was 1.6 ± 0.3 mg kg^−1^, and for MIX, it was 0.9 ± 0.1 mg kg^−1^.

**Fig. 2:**
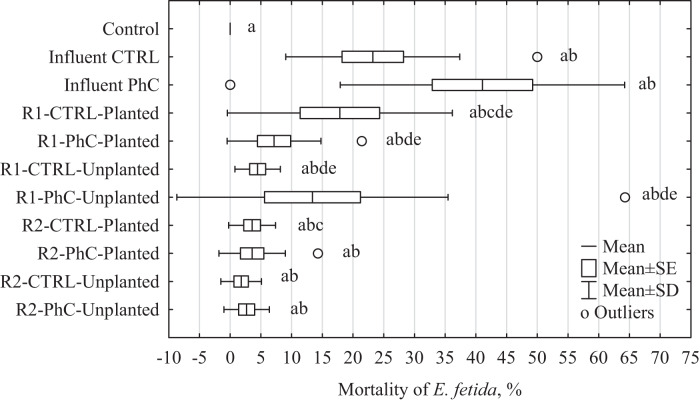
Effects of CW wastewater on *E. fetida* mortality; Control: distilled water; Influent CTRL: control wastewater; Influent PhC: wastewater enriched by DCF and SMX; R1: wastewater from rack 1; R2: wastewater from rack 2; CTRL: control wastewater (without DCF and SMX); PhC: tested wastewater (enriched by DCF and SMX); Planted: wastewater from planted columns (planted by *Miscanthus giganteus*); Unplanted: wastewater from columns without plants; a: statistically significant differences in relation to control samples; b: statistically significant differences between the toxicity levels of the wastewater influent and effluent; c: statistically significant differences between the toxicity of wastewater from racks with different frequencies of sewage dosing; d: statistically significant differences between the toxicity of control wastewater and sewage containing pharmaceuticals; e: statistically significant differences between the toxicity of wastewater from columns with and without plants; Mann–Whitney *U* test, *p* < 0.05

A high toxic effect of raw wastewater containing DCF and SMX (Influent PhC) was observed (Fig. 2 and Supplementary Table SI.6). In samples exposed to raw wastewater containing PhC, the mortality of adult earthworms (after 28 days) was 41.1% ±9.9%. Additionally, the earthworms that had survived the experiment showed significant damage (Supplementary Fig. SI.2). Similar results for MIX were obtained in preliminary tests, where mortality at the highest concentration (4 mg kg^−1^) was 100%. The compounds analyzed had a significant impact on the fertility of *E. fetida*. Raw wastewater (influent CTRL) reduced the number of juveniles by 56.4 ± 2.9% (Mann–Whitney *U* test, *p* = 0.0008), while wastewater containing DCF and SMX reduced this number by 75.0 ± 12.7% (Mann–Whitney *U* test, *p* = 0.0004). The treated wastewater showed a lower toxicity for *E. fetida*, especially in the case of the effluent from Rack 2, where the mean mortality was only 2.9%, while for Rack 1, the mean mortality was 12.6%. However, unlike reproduction tests, only for CTRL-Planted, a statistically significant effect of the applied wastewater dosing system on *E. fetida* mortality was observed (Mann–Whitney *U* test, *p* = 0.03). The presence of PhC and plants had a significant effect on toxicity only in rack 1 wastewater (Mann–Whitney *U* test, for PhC *p* = 0.02 and for plants *p* = 0.005).

**Fig. 3:**
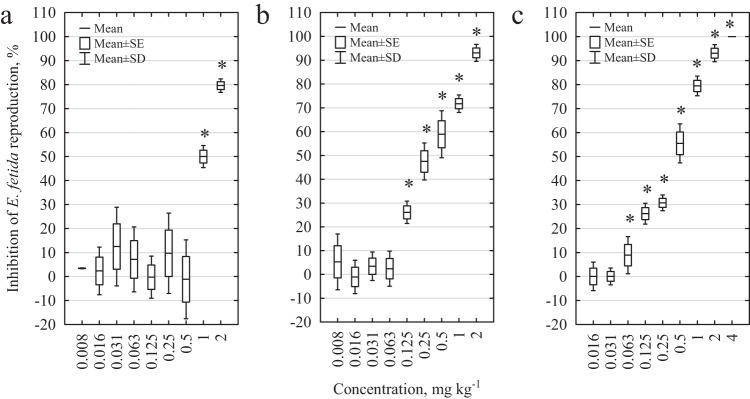
Effects of pharmaceuticals and their mixture on the reproduction of *E. fetida*. **a** effect of DCF, **b** effect of SMX, and **c** effect of DCF and SMX mixture. * statistically significant differences compared to the control sample; Student’s *t*-test, *α* = 0.05

The tested pharmaceuticals (DCF and SMX) and their binary mixture (MIX) clearly inhibited the reproduction of *E. fetida* (Fig. 3). In the case of SMX, statistically significant differences were observed even at low concentration (0.125 mg kg^−1^ and above, Student’s *t*-test, *p* = 0.001). In the case of DCF, only concentrations of 1 and 2 mg kg^−1^ effected the fertility of *E. fetida* (for 1 mg kg^−1^ *p* = 0.0001 and for 2 mg kg^−1^ *p* = 0.00001, Student’s *t*-test). For MIX, statistically significant differences were observed at a concentration of 0.063 mg kg^−1^ and above (Student’s *t*-test, *p* = 0.001). The EC_50_ determined for DCF was 1.2 ± 0.2 mg kg^−1^; for SMX, it was 0.4 ± 0.1 mg kg^−1^, and for MIX, it was 0.3 ± 0.1 mg kg^−1^.

**Fig. 4:**
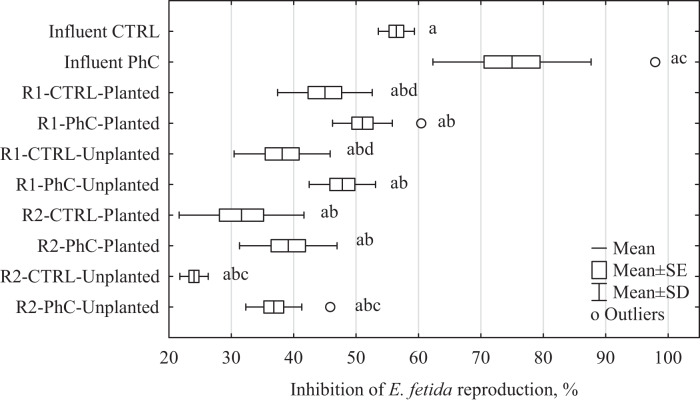
Effects of CW wastewater on *E. fetida* reproduction; Influent CTRL: control wastewater; Influent PhC: wastewater enriched by DCF and SMX; R1: wastewater from Rack 1; R2: wastewater from Rack 2; CTRL: control wastewater (without DCF and SMX); PhC: tested wastewater (enriched by DCF and SMX); Planted: wastewater from planted columns (planted by *Miscanthus giganteus*); Unplanted: wastewater from columns without plants; a: statistically significant differences between toxicity levels of wastewater influent and effluent; b: statistically significant differences between the toxicity of wastewater from racks with different frequencies of sewage dosing; c: statistically significant differences between toxicity of control wastewater and sewage containing pharmaceuticals; d: statistically significant differences between the toxicity of wastewater from columns with and without plants; Mann–Whitney *U* test, *p* < 0.05

The influence of CW wastewater on *E. fetida* reproduction was investigated for treated and raw sewage. Inhibition of fertility in relation to control conditions (after 56 days of incubation) is presented in Fig. 4 and Supplementary Table SI.5. The highest inhibition of reproduction was observed for raw wastewater samples (for Influent CTRL 56.4 ± 2.9% and for Influent PhC 75.0 ± 12.7%). The treated wastewater showed lower toxicity but a high inhibition of fertility, from 24 to 51% (rack 1: 45.5% on average, rack 2: 32.9% on average). There were statistically significant differences between the toxicity levels of wastewater from different wastewater dosing systems (Supplementary Table SI.5, Mann–Whitney *U* test, *p* < 0.05). Wastewater detoxification was more efficient in Rack 2 (sewage was administered twice a week in a 2.5 L volume). Surprisingly, neither the presence of PhC in sewage nor *M. giganteus* in CWs considerably influenced the toxicity of treated wastewater.

### Oxidative stress in *E. fetida*

**Fig. 5:**
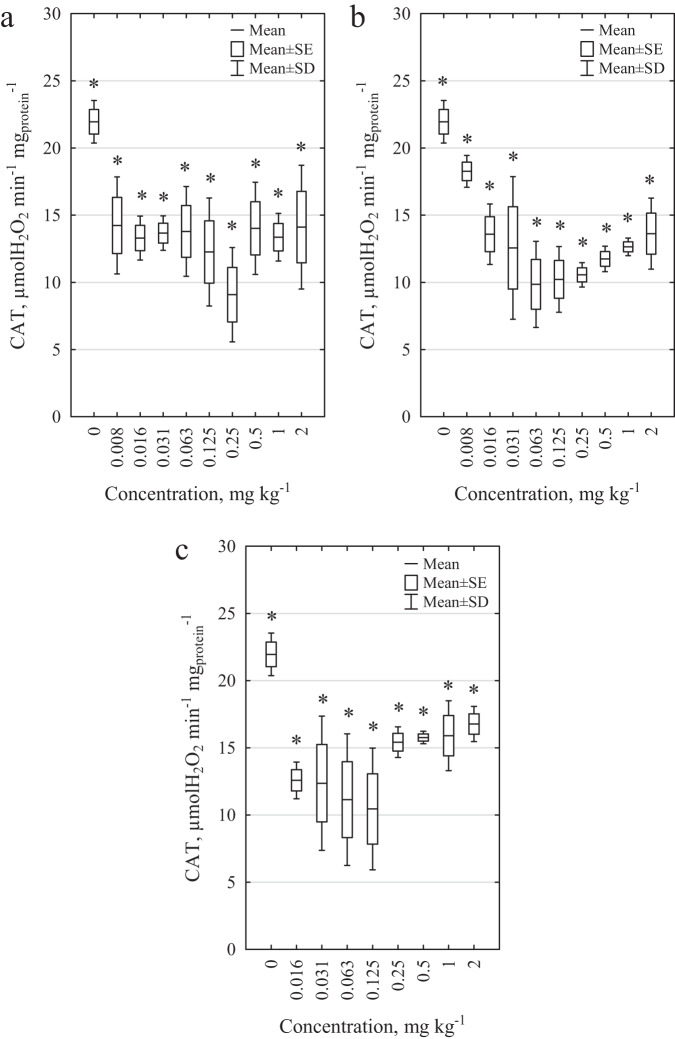
Effects of pharmaceuticals and their mixture on catalase activity in homogenates of *E. fetida*. **a** effect of DCF, **b** effect of SMX, and **c** effect of DCF and SMX mixture. * statistically significant differences in relation to the control; Student’s *t*-test, *α* = 0.05

The results of CAT activity in *E. fetida* earthworm homogenates exposed to DCF, SMX, and MIX are presented in Fig. 5. In the case of MIX, it was not possible to determine the activity of antioxidant enzymes at the highest concentration tested concentration (4 mg kg^−1^), due to the high mortality rate of earthworms after 28 days of the test (mortality at 4 mg kg^−1^ was 100%). For this reason, the enzyme activity was only determined to a concentration of 2 mg kg^−1^.

The presence of DCF and SMX caused a decrease in CAT activity in *E. fetida*. The highest effect of DCF was observed at a concentration of 0.25 mg kg^−1^ (decrease in enzymatic activity by 58.6%). A similar effect was observed for SMX. The highest effect was observed at a concentration of 0.063 mg kg^−1^ (decrease by 55.1%). It should be noted that the greatest effect (decrease in CAT activity) was observed not for the highest concentration analyzed concentration (2 mg kg^−1^) but for 0.25 mg kg^−1^ in the case of DCF and 0.063 mg kg^−1^ for SMX. These concentrations elicited the highest responses from the test organisms. In the case of MIX, the highest effect was observed at 0.125 mg kg^−1^ (52.4%). At concentrations from 0.25 to 2 mg kg^−1^, CAT activity ranged from 15.4 to 16.8 µmol H_2_O_2_ min^−1^ mg_protein_^−1^.

The effects on CAT activity in *E. fetida* exposed to CW wastewater are presented in Table 3 and Supplementary Table SI.6. A statistically significant decrease in CAT activity was observed for samples treated with the analyzed wastewater. The highest decrease in activity was observed in relation to control conditions for Influent PhC (46.3%). It is also worth noting that no influence of technological parameters on the activity of this enzyme was observed (Supplementary Table SI.6, Student’s *t*-test, *α* = 0.05). Furthermore, no differences in CAT activity were observed between raw and treated wastewater (Supplementary Table SI.6, Student’s *t*-test, *α* = 0.05). The only exception was sewage from columns planted with *M. giganteus* and spiked with pharmaceuticals.

**Table 3:** Decrease in CAT activity as a result of the analyzed wastewater

Columns	dCAT^a^ (%)
Influent CTRL	33.2
Influent PhC	46.3
R1-CTRL-Planted	21.5
R1-PhC-Planted	29.4
R1-CTRL-Unplanted	27.7
R1-PhC-Unplanted	34.4
R2-CTRL-Planted	17.6
R2-PhC-Planted	29.4
R2-CTRL-Unplanted	25.0
R2-PhC-Unplanted	34.7

^a^The decrease in CAT activity (dCAT) was calculated based on the enzyme activity caused by wastewater in relation to the control conditions according to the following equation: ${\rm{dCAT}}\left( \% \right) = \frac{{A_{{\rm{CTRL}}} - A_{{\rm{wastewater}}}}}{{A_{{\rm{CTRL}}}}} \cdot 100$

**Fig. 6:**
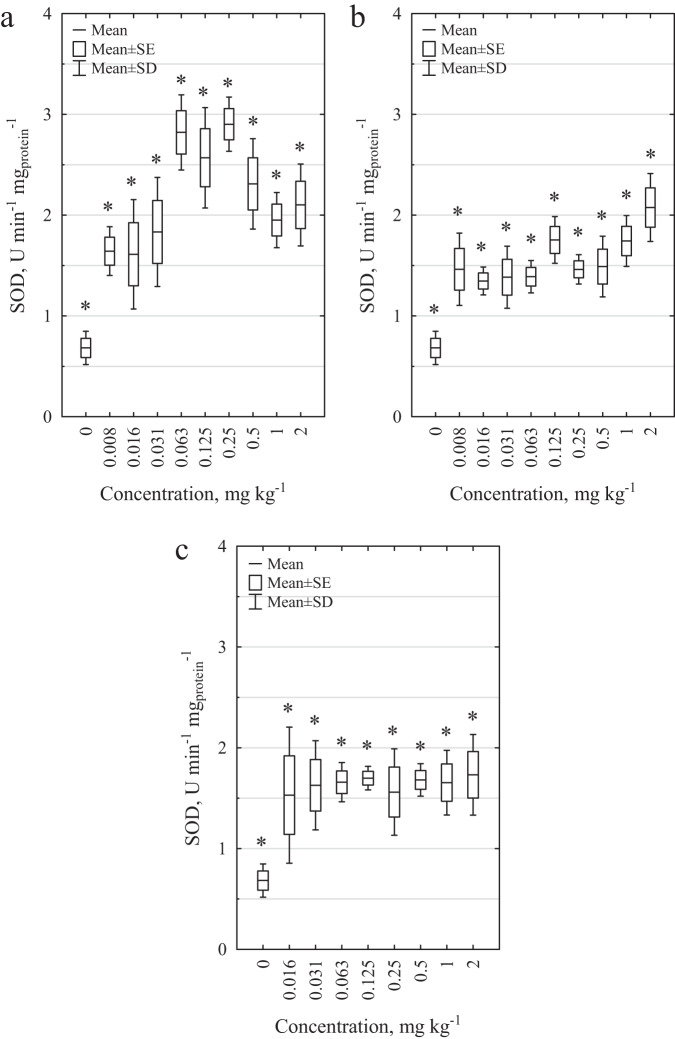
Effects of pharmaceuticals and their mixture on the activity of SOD in homogenates of *E. fetida*. **a** effect of DCF, **b** effect of SMX, and **c** effect of DCF and SMX mixture. * Statistically significant differences in relation to the control; Student’s *t*-test, *α* = 0.05

In the case of SOD (Fig. 6), all the solutions tested caused an increase in enzymatic activity. For DCF, the highest effect in relation to the control conditions was observed at 0.25 mg kg^−1^ (by 324.9%), whereas for SMX, the highest SOD activity was observed at 2 mg kg^−1^ (203.9%). However, in the case of MIX, the SOD activity remained constant and was higher than the control level (with an average increase of 140.5%). Enzymatic activity was 1.5–1.7 U min^−1^ mg_protein_^−1^ in all tested MIX concentrations.

In the case of SOD, as in the preliminary tests, an increase in enzymatic activity was observed under the influence of wastewater (Table 4 and Supplementary Table SI.7). The highest increase in SOD activity was observed for wastewater from R1-PhC-planted columns (by more than 200%). Additionally, for Influent PhC, an increase of more than 160% in SOD activity was observed. Similar to the analysis of CAT activity, plants had no effect on SOD activity (Supplementary Table SI.7, Mann–Whitney *U* test, *p* < 0.05). However, statistically significant differences were observed between the frequency of wastewater dosing in the case of wastewater from columns planted with *M. giganteus* (Supplementary Table SI.7, Mann–Whitney *U* test, *p* < 0.05). A more pronounced detoxification of sewage in CW was observed for Rack 2, where wastewater was distributed five times a week in a volume of 1 L.

**Table 4:** Increase in SOD activity as a result of analyzed wastewater

Columns	iSOD^a^ (%)
Influent CTRL	81.6
Influent PhC	164.4
R1-CTRL-Planted	112.9
R1-PhC-Planted	212.2
R1-CTRL-Unplanted	46.6
R1-PhC-Unplanted	108.4
R2-CTRL-Planted	34.8
R2-PhC-Planted	59.3
R2-CTRL-Unplanted	39.3
R2-PhC-Unplanted	77.1

^a^The increase in SOD activity (iSOD) was calculated based on the enzyme activity caused by wastewater in relation to the control conditions according to the following equation: ${\rm{iSOD}}\left( \% \right) = \frac{{A_{{\rm{wastewater}}} - A_{{\rm{CTRL}}}}}{{A_{{\rm{CTRL}}}}} \cdot 100$

## Discussion

### Effects of microcontaminants on mortality and reproduction in *E. fetida*

Micropollutants that occur in the natural environment can pose a serious threat to living organisms. One of the most important factors that affect the toxicity of xenobiotics is their physicochemical properties. The properties of the pharmaceuticals selected for these tests (Supplementary Table SI.1) may indicate their ability to accumulate in the soil. In our study, DCF, with logK_OW_ (4.51) and pKa (4.15) relative to the pH of artificial soil (5.8), showed toxicity toward *E. fetida* only at concentrations of 1 mg kg^−1^ and higher (Fig. 1a). This could be due to the sorption of DCF on soil particles; as a result, DCF was not easily available to earthworms. On the contrary, SMX is characterized by a lower logK_OW_ (0.89) and pKa (5.6–5.7), close to the soil pH, making it more readily available to soil organisms.

In studies carried out on pure pharmaceuticals, the toxicity of the tested drugs was observed on model organisms. DCF and SMX caused a statistically significant increase in earthworm mortality at a concentration of 0.5 mg kg^−1^, while the mixture of tested drugs showed an effect on this parameter already at a concentration of 0.25 mg kg^−1^ (meaning a concentration of DCF of 0.125 mg kg^−1^ and a concentration of SMX of 0.125 mg kg^−1^, respectively). Currently, DCF and SMX are detected in soil at much lower concentrations (0.2 mg kg^−1^ for DCF and 0.05 mg kg^−1^ for SMX, respectively), but the ever-increasing use of drugs by humans and for veterinary purposes, as well as decreasing water resources, can lead to an increase in the amount of micropollutants detected in the environment in the near future (Ashfaq et al. 2019; Cycoń et al. 2019).

The high mortality of earthworms, especially in experiments with a mixture of the tested pharmaceuticals, had an impact on the number of offspring. Pino et al. (2015) in his studies showed that the LC_50,14 days_ for DCF was 90.49 mg kg^−1^, whereas for SMX, it was higher than 4000 mg kg^−1^. In our study, the LC_50_ values were determined for adult earthworms incubated for 28 days (LC_50,28 days_ for DCF was 3.4 ± 0.3 mg kg^−1^ and for SMX 1.6 ± 0.3 mg kg^−1^); thus, a higher toxicity was observed due to the longer contact time of the test organisms with the test contaminants. As under natural conditions, organisms can have a longer contact period with pollutants, so it is important to assess long-term effects, which are usually caused by much lower concentrations of the analyzed micropollutants.

The situation is even more serious in the case of the effects of the test drugs on the reproduction of *E. fetida* earthworms. DCF caused a statistically significant inhibition in *E. fetida* reproduction at a concentration of 1 mg kg^−1^; however, in case of SMX negative effect was observed at 0.125 mg kg^−1^. In case of MIX, a statistically significant reduction in the number of juveniles was already observed at a concentration of 0.063 mg kg^−1^ (meaning a concentration of DCF of 0.031 mg kg^−1^ and a concentration of SMX of 0.031 mg kg^−1^, respectively). The values obtained for the mixture of pharmaceuticals are lower than the concentrations detected in the natural environment, which means that they pose a serious and real threat to living organisms.

As we have shown in our previous studies (Drzymała and Kalka 2020), a mixture of DCF and SMX may induce additive or synergistic effects toward aquatic organisms. In the case of the mortality and reproduction tests of *E. fetida*, enhanced MIX toxicity was observed. The interactions occurring in the pharmaceutical mixture were determined on the basis of the mixture toxicity index (MTI) value. The interactions between the mixture components, determined based on the MTI values, are classified in Supplementary Table SI.4 (Nagata et al. 2008).

For both *E. fetida* mortality (MTI = 3.29) and reproduction (MTI = 3.41), the toxicity index was higher than 1, suggesting a synergistic effect of the mixture. Data on the toxic effects of mixtures on soil invertebrates are rare. However, Yu et al. (2019) showed that cadmium has synergistic effects in binary, ternary, quaternary and quinquenary mixtures with pesticides (atrazine, chlorpyrifos, lambda-cyhalothrin, and abamectin) toward *E. fetida*. Chen et al. (2014) proved that pesticides (butachlor, imidacloprid, and chlorpyrifos) in binary mixtures had antagonistic or additive effects on *E. fetida*. The synergistic effect of mixtures poses a serious risk to the environment and it is difficult to predict the complex mechanisms of interaction of various pollutants found in mixtures that can be introduced into the environment. However, experiments performed only for individual contaminants may underestimate the actual toxic effects, which can only be observed in multiple mixtures in the environment.

The studies carried out on wastewater from the constructed wetlands showed that the greatest impact on the mortality and reproduction of the earthworm *E. fetida* had raw sewage, especially those enriched with the tested pharmaceuticals. Wastewater containing DCF and SMX entering the treatment plant resulted in 41% mortality of the test organisms and 75% inhibition of reproduction.

It is also worth paying attention to the aspect of effectiveness of the proposed constructed wetland system. When assessing the effectiveness of the wastewater treatment process, detoxification is often assessed, according to the definition proposed by Fortney et al. (2018), it is a set of processes aimed at identifying, neutralizing, and eliminating toxic substances and their decomposition products. Thus, detoxification can be defined as the process of reducing the toxicity of a substance or wastewater. All the experiments conducted showed that wastewater after treatment in the constructed wetland system was less harmful to *E. fetida* organisms than raw sewage, therefore, it can be clearly stated that the proposed treatment system reduced the toxicity of sewage. However, the determined harmfulness of the leachate was so high that the wastewater after treatment should not be discharged directly into the natural environment. Their introduction would require the use of additional treatment methods, which would undoubtedly increase the cost of the process itself (Singh 2021).

The dosing system used may be of key importance in reducing the toxicity of wastewater in the case of a constructed wetland system. In our experiment, differences were observed in reproduction inhibition in *E. fetida* treated with wastewater from systems with different types of supply (Fig. 4). For columns with sewage distributed in five batches per week at a volume of 1 L (rack 2), a lower toxicity of treated wastewater was observed (inhibition of reproduction by 32.9%) compared to columns with sewage dosed twice per week at a volume of 2.5 L (inhibition of reproduction by 45.5%). Similar results were observed in our previous study (Drzymała et al. 2022), where the effect of the frequency of wastewater dosing on the toxicity toward three aquatic bioindicators, namely *Aliivibrio fischeri*, *Daphnia magna*, and *Lemna minor*, was observed. However, to our knowledge, this relationship was observed for the first time in long-term reproduction tests. Based on our previous research (Drzymała et al. 2022), it can be concluded that the presence of plants is not a key factor in wastewater treatment. The operating parameters of the selected wastewater treatment system, such as the frequency and volume of wastewater, the plants used, the column filling used, can have a key impact on the effectiveness of the treatment process (He et al. 2021; Hu et al. 2021; Sochacki et al. 2021).

Tests based on *E. fetida* earthworms are a highly reliable source of information on the toxicity of various compounds. The endpoint of the *E. fetida* toxicity tests may be mortality, growth retardation, or reproduction inhibition, making it a universal organism for testing contaminants (Zhang et al. 2020; Zhu et al. 2020). Studies using earthworms as a model organism largely focused on pollutants directly or intentionally introduced into the soil, such as pesticides (Pescatore et al. 2021; Teng et al. 2022; Yang et al. 2017b), heavy metals (Sheng et al. 2021; Yu et al. 2019), or mixtures thereof (Li et al. 2019, 2020a; Uwizeyimana et al. 2018; Yu et al. 2019). However, there is a lack of information on contaminants that can reach the soil as a result of spreading manure containing pharmaceuticals used in, for example, veterinary medicine. Similarly, toxicity tests on soil invertebrates based on determining the detoxification of xenobiotics during wastewater treatment are scarce. Wang and Lemley (2003) showed that earthworms can be good bioindicators for the detoxification of insecticides in the soil. Toxicity tests based on changing earthworm weight showed that the proposed treatment using the Fenton process was highly effective against six carbamate insecticides (Wang and Lemley 2003). Also, de Moura Lisbôa et al. (2021) showed that the discharge of sanitary wastewater can negatively impact *Eisenia andrei* earthworms. Studies have also investigated the behavior and reproduction of earthworms, the activity of enzymes (acetylcholinesterase, catalase, and superoxide dismutase) and the level of malondialdehyde. The results confirmed that sanitary sewage affected reproduction and behavior and induced oxidative stress in earthworms.

Earthworms play an important role in the introduction of organic matter and the supply of nutrients to plants, making them good soil bioindicators in wastewater treatment, land reclamation, remediation, or the recycling of organic waste. Furthermore, *E. fetida* earthworms might be used to intensify wastewater treatment processes in constructed wetlands. Studies have shown that the addition of earthworms to vertical flow constructed wetlands can increase the substrate nitrification potential in both vegetated and unvegetated systems, although a greater effect on nitrification was observed in vegetated CWs (Xu et al. 2013). In another study, the presence of earthworms stimulated the growth of wetland plants (plant height, leaf width and stem diameter) (Lavrnić et al. 2019). In wastewater treatment systems, earthworms also affect chemical parameters such as chemical oxygen demand, ammonium nitrogen, total nitrogen, and total phosphorus (Singh et al. 2019).

The presence of pharmaceutical substances in the natural environment is a serious problem that poses a risk to aquatic and soil organisms. The release of treated, potentially harmful, sewage into the environment can have serious long-term effects on nontarget organisms. As selective toxicity studies of wastewater discharged into the environment cannot adequately show the effects of sewage on living organisms, it is necessary to place an emphasis on chronic effects, such as effects on reproduction, fertility, genotoxicity, and oxidative stress. These are highly sensitive indicators of the influence of pollutants, even at low concentrations or in complex mixtures.

### Effects of microcontaminants on oxidative stress in *E. fetida*

Various biochemical responses of organisms to environmental stress are considered as early warning indicators of environmental pollution (Song et al. 2009). Examples of such biomarkers are antioxidant enzymes and their activities. The main function of the antioxidant system is to protect cells from the harmful effects of reactive oxidative species (ROS). Under normal conditions, cells can reduce ROS to water through the electron transport chain. However, under stressful conditions, such as the presence of xenobiotics, antioxidant enzymes such as SOD and CAT can be activated (Wu et al. 2011). Of these, SOD activation, which catalyzes the reaction of the dismutation of the superoxide radical to water and oxygen peroxide, is the first defense mechanism activated in cells. An increase in the activity of this enzyme indicates an increase in O_2_^-^ production. Hydrogen peroxide, which is a reaction product catalyzed by SOD, is also a substrate of the CAT reaction. Inhibition of this enzyme can result from changes in CAT biosynthesis, inactivation, or damage to the enzyme structure by ROS. Excess H_2_O_2_ can accumulate in cells and induce the formation of oxidative damage, causing changes in genetic material, consequently leading to cell or organism death (Wang et al. 2016). The activity of antioxidant enzymes is not constant and changes over time. Most often, an increase in CAT activity is observed when the organism encounters low levels of stress, whereas a decrease in the activity of this enzyme can be seen under severe stress (Song et al. 2012).

For all pharmaceuticals tested (DCF, SMX and MIX), a statistically significant decrease in CAT activity and an increase in SOD activity, even at low concentrations, was observed, indicating the influence of the tested drugs and their mixture on the activity of antioxidant enzymes. The decrease in CAT activity may be due to the toxic effects of pharmaceuticals on the test organism and the effect of ROS. When the amount of ROS exceeds the cell’s ability to remove them, they become inhibitors of antioxidant enzymes. When the oxidative stress induced by xenobiotics is high, the organism’s defense processes can be inhibited (Lin et al. 2010; Schreck et al. 2008).

Changes in the activities of antioxidant enzymes with respect to control conditions always indicate adverse effects on the test organisms. Increased SOD activity reflects activation of cell defense mechanisms and thus indicates the presence of oxidative stress. A decrease in CAT activity may indicate a highly toxic effect of the pharmaceuticals tested. Here, the induced oxidative stress was high enough to inhibit some defense mechanisms, which can lead to cell dysfunction and apoptosis (Gao et al. 2008). The increase in *E. fetida* mortality in the highest concentrations tested confirms these conclusions.

High standard deviations of CAT activity indicate different ages of the test organisms. In our study, earthworms from our own breeding program were used, with reproducibility ability and body weight between 300 and 600 mg (according to the OECD (2016) standard). However, according to the findings of Saint**-**Denis et al. (1998), the activity of CAT in older individuals is higher than that in younger organisms. Similar conclusions have been obtained by Ravi Kiran and Aruna (2010). The authors also analyzed CAT activity in *Eudrilus eugeniae*, and an inverse correlation has been found for SOD activity; the older the test organisms, the lower the activity of this enzyme (Ravi Kiran and Aruna 2010).

Similarly, as in the case of pure pharmaceuticals, wastewater from the wetland treatment plant caused a decrease in CAT activity and an increase in SOD activity in cells from test organisms. The highest effect in the case of CAT activity was caused, similar to the mortality and reproduction test, by raw effluents containing DCF and SMX (a decrease of 46% compared to the control conditions). However, in the case of SOD activity, the dependencies are not so obvious. The highest increase in the activity of this enzyme was observed in leachate (R1-PhC-Planted, increased by 212%). This may indicate that other factors can be responsible for the oxidative stress caused in *E. fetida* earthworms than those that led to mortality or reproduction changes in the test organisms. The reason for the higher activity of SOD in leachates may be the products of the decomposition of pharmaceuticals present in the effluents or the greater amount of superoxide radical resulting from the ongoing purification processes (Sharif et al. 2016).

The exposure time of the test organisms to wastewater may also be an important factor impacting enzyme activity. In our study, the earthworms of *E. fetida* were exposed to the analyzed sewage for 28 days. Cao et al. (2017) observed that after just 14 days, the activity of CAT may be inhibited by the action of eutrophic water containing cyanobacteria toxins. It has been suggested that a decrease in CAT activity may be caused by ROS accumulation due to the long incubation period. Similar results have been obtained by Schreck et al. (2008). Studies conducted on the nocturnal earthworm *Aporrectodea caliginosa nocturna*, using six pesticides, showed an increase in CAT activity after 3 days of exposure. However, after 14 days, inhibition of enzymatic activity was observed. The authors of the article suggested that inhibition of CAT activity is caused by excessive ROS, and thus a strong toxic effect, inhibiting CAT metabolism (Schreck et al. 2008). Mkhinini et al. (2019a) found results similar to those observed in the present study. These authors also suggested that the adaptation process may be useful during experiments with long exposure of model organisms to toxic compounds (Mkhinini et al. 2019a). However, when analyzing the toxicity of five agricultural soils irrigated with treated wastewater towards *Eisenia andrei*, the same authors observed an increase in CAT activity after 14 days of exposure (Mkhinini et al. 2019b).

Antioxidant enzyme activity can be a useful tool to determine the early effects of the substances analyzed on model organisms. On the basis of the assessment of the activities of antioxidant enzymes, we can detect changes that are undetectable in other tests (e.g., reproduction or mortality tests). Due to the large variability in the composition of wastewater over time, these tests are rarely used to determine sewage toxicity; however, they may be helpful in assessing its harmfulness.

We highlight the need to pay attention to the presence of micropollutants introduced into the soil with wastewater. Long-term exposure of life-supporting organisms, responsible for soil formation processes, to such pollutants can cause irreversible damage and disturb the balance of the soil ecosystem.

## Conclusions

Micropollutants, such as pharmaceuticals, that are present in the natural environment can severely affect soil organisms. Based on our findings, both diclofenac and sulfamethoxazole can impact the mortality and reproduction of *E. fetida* earthworms. Additionally, the binary mixture of the PhCs mentioned above had a synergistic toxic effect on soil invertebrates. Wastewater from vertical-flow constructed wetland systems can also negatively affect soil organisms, which was the case for treated and untreated wastewater in our study. Moreover, the effect of technical parameters of wastewater treatment is described for the first time in a long-term reproduction test. The frequency of wastewater dosing to the CW system determined the observed toxicity of treated sewage. When sewage was dispensed five times a week in a volume of 1 L, the toxicity was lower. Other technical parameters, such as the presence of *M. giganteus*, were not essential to the purification process. In addition, antioxidant enzyme activity can be a useful indicator for soil contaminants. Micropollutants are present in the soil at low concentrations, often leading to oxidative stress, which is the first defense mechanism of organisms. The activities of antioxidant enzymes (such as catalase or superoxide dismutase) can indicate an emerging problem before more serious effects occur in model organisms (such as death). The investigated pharmaceuticals and wastewater caused a decrease in CAT activity and an increase in SOD activity and therefore disturbed the homeostasis of *E. fetida*, causing oxidative stress.

The use of earthworms *E. fetida* as a bioindicator of pollution allows estimating the effects on the entire population of soil organisms. The presence of microcontaminants in the soil environment should not be underestimated. Further studies should focus on interactions among various pollutants and on monitoring the origin of wastewater sludge or effluent used as fertilizer or for agricultural irrigation.

### Supplementary Information

Supplementary Information
