# Supplementary material for: Effects of diclofenac, sulfamethoxazole, and wastewater from constructed wetlands on Eisenia fetida: impacts on mortality, fertility, and oxidative stress
Source: Ecotoxicology. 2023 Aug 26;32(7):858–73. doi: 10.1007/s10646-023-02690-3 (PMC10533613; doi:10.1007/s10646-023-02690-3)
Supplement: Supplementary file 1 — Supplementary Information [file 10646_2023_2690_MOESM1_ESM.docx]

**Supplementary Information**

**Effects of diclofenac, sulfamethoxazole, and wastewater from constructed wetlands on *Eisenia fetida*: impacts on mortality, fertility, and oxidative stress**

Drzymała Justyna^1*^, Kalka Joanna^2^

^1^ Silesian University of Technology, The Biotechnology Centre, Gliwice, Poland

^2^ Silesian University of Technology, Environmental Biotechnology Department, Faculty of Energy and Environmental Engineering, Gliwice, Poland

^*^Corresponding author: Silesian University of Technology, The Biotechnology Centre, Krzywoustego 8, 44-100 Gliwice, Poland, tel. +48 32 400 30 90, e-mail: justyna.drzymala@polsl.pl

**Table SI.1** The basic properties of diclofenac (DCF) and sulfamethoxazole (SMX)

| **Compound** | **CAS number** | **Molecular formula** | **Molar mass,**  **g mol^-1^** | **pK_a_^1^** | **logK_OW_^1^**  **(pH 8)** |
| --- | --- | --- | --- | --- | --- |
| DCF | 15307-86-5 | C_14_H_11_Cl_2_NO_2_ | 296.15 | 4.15 | 4.51 |
| SMX | 723-46-6 | C_10_H_11_N_3_O_3_S | 253.28 | 5.6 – 5.7 | 0.89 |

^1^ Pal et al. 2010


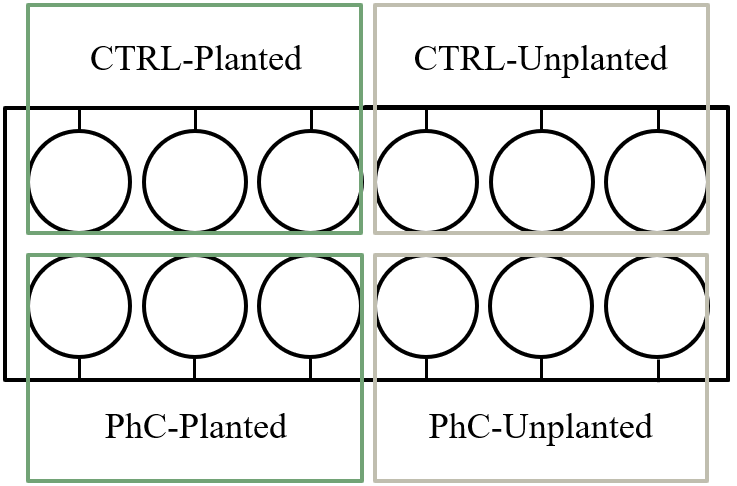


**Fig. SI.1** The scheme of experimental system

**Table SI.2** Removal efficiency of diclofenac (DCF), sulfamethoxazole (SMX), total organic carbon (TOC), and ammonium nitrogen (N-NH_4_) in constructed wetlands

| **Columns description** | **Removal efficiency, %^*^** | | | |
| --- | --- | --- | --- | --- |
|  | DCF | SMX | TOC | N-NH_4_ |
| R1-CTRL-Planted | – | – | 87.8 ±3.93 | 19.9 ±7.3 |
| R1-PhC-Planted | 68.8 ±8.2 | 79.1 ±4.3 | 87.3 ±1.94 | 18.0 ±11.4 |
| R1-CTRL-Unplanted | – | – | 90.8 ±1.08 | 39.3 ±10.3 |
| R1-PhC-Unplanted | 13.7 ±16.3 | 78.9 ±22.5 | 90.0 ±0.97 | 22.9 ±12.6 |
| R2-CTRL-Planted | – | – | 93.3 ±1.43 | 45.8 ±11.9 |
| R2-PhC-Planted | 86.8 ±9.7 | 98.0 ±0.8 | 92.3 ±1.12 | 58.9 ±10.0 |
| R2-CTRL-Unplanted | – | – | 93.7 ±1.07 | 29.1 ±8.3 |
| R2-PhC-Unplanted | 76.6 ±9.4 | 97.4 ±0.7 | 94.1 ±0.59 | 58.3 ±4.6 |

^*^The removal efficiency (R) was calculated based on the influent and effluent concentrations to the following equation: $R(\%)= \frac{C_{influent}-C_{effluent}}{C_{influent}} \cdot100$

**Table SI.3** The composition of artificial soil

| Component | Description | % of dry weight |
| --- | --- | --- |
| sphagnum peat | pH in range 5.5 – 6.0, dried to measured moisture content | 10% |
| kaolin clay | kaolinite content above 30% | 20% |
| calcium carbonate | to obtain pH in range 5.5 – 6.5 | 0.3% – 1% |
| quartz sand | sand with more than 50% of particles size between 50 – 200 μm | app. 70% |

**Table SI.4** Classification of interactions in a mixture based on mixture toxicity index (MTI) values

| **MTI [–]** | **Interactions** |
| --- | --- |
| MTI < 0 | antagonistic effect |
| MTI = 0 | no-additive effect |
| 0 < MTI < 1 | partial additive effect |
| MTI = 1 | additive effect |
| MTI > 1 | synergistic effect |

**Table SI.5** Results of mortality and reproduction tests toward *E. fetida*

| **Columns** | **Mortality, %** | **Inhibition of reproduction, %** |
| --- | --- | --- |
| CTRL | 0.0 ±0.0 | – |
| Influent CTRL | 23.2 ±6.4 | 56.4 ±2.9 |
| Influent PhC | 41.1 ±9.9 | 75.0 ±12.7 |
| R1-CTRL-Planted | 9.8 ±3.7 a | 45.0 ±7.6 |
| R1-PhC-Planted | 22.5 ±5.8 b | 51.0 ±4.8 |
| R1-CTRL-Unplanted | 4.5 ±3.7 c | 38.1 ±7.7 |
| R1-PhC-Unplanted | 13.4 ±5.1 d | 47.8 ±5.3 |
| R2-CTRL-Planted | 3.6 ±3.8 a | 31.6 ±10.0 |
| R2-PhC-Planted | 3.6 ±3.8 b | 39.1 ±7.8 |
| R2-CTRL-Unplanted | 1.8 ±3.3 c | 24.0 ±2.3 |
| R2-PhC-Unplanted | 2.7 ±3.7 d | 36.8 ±4.5 |

The p-values in Mann-Whitney U test in case of mortality of *E. fetida* to obtained for comparing columns operating in different sewage dosing systems: a = 0.024, b = 0.007; c = 0.001, d = 0.006;


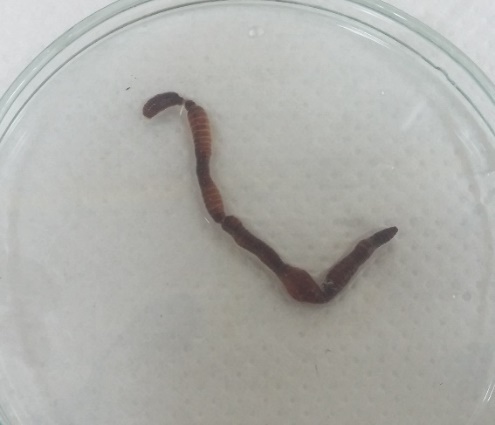


**Fig. SI.2** The damaged earthworms under the influence of raw sewage containing pharmaceuticals

**Table SI.6** Results of catalase (CAT) activity in *E. fetida* exposed to wastewater from constructed wetlands

| **Columns** | **CAT activity**  µmol H_2_O_2_ min^-1^ mg_protein_^-1^ |
| --- | --- |
| CTRL | 35.28 ±4.51 ^a^ |
| Influent CTRL | 23.58 ±5.84 ^a^ |
| Influent PhC | 18.95 ±5.64 ^ab^ |
| R1-CTRL-Planted | 27.69 ±5.04 ^a^ |
| R1-PhC-Planted | 24.91 ±4.85 ^ab^ |
| R1-CTRL-Unplanted | 25.49 ±5.49 ^a^ |
| R1-PhC-Unplanted | 23.13 ±4.94 ^a^ |
| R2-CTRL-Planted | 29.05 ±4.74 ^ac^ |
| R2-PhC-Planted | 24.89 ±2.63 ^abc^ |
| R2-CTRL-Unplanted | 26.46 ±3.45 ^a^ |
| R2-PhC-Unplanted | 23.03 ±3.58 ^a^ |

^a^ statistically significant differences in relation to the control samples, ^b^ statistically significant differences between toxicity of wastewater influent and effluent, ^c^ statistically significant differences between toxicity of control wastewater and sewage containing pharmaceuticals; Student's t test, α=0.05

**Table SI.7** Results of superoxide dismutase (SOD) activity in *E. fetida* exposed to wastewater from constructed wetlands

| **Columns** | **SOD activity**  U min^-1^ mg_protein_^-1^ |
| --- | --- |
| CTRL | 0.27 ±0.04 ^a^ |
| Influent CTRL | 0.50 ±0.11 ^ab^ |
| Influent PhC | 0.73 ±0.20 ^ab^ |
| R1-CTRL-Planted | 0.59 ±0.28 ^ac^ |
| R1-PhC-Planted | 0.86 ±0.41 ^ac^ |
| R1-CTRL-Unplanted | 0.40 ±0.07 ^a^ |
| R1-PhC-Unplanted | 0.57 ±0.22 ^a^ |
| R2-CTRL-Planted | 0.37 ±0.05 ^abcd^ |
| R2-PhC-Planted | 0.44 ±0.06 ^abcd^ |
| R2-CTRL-Unplanted | 0.38 ±0.11 ^ab^ |
| R2-PhC-Unplanted | 0.49 ±0.20 ^ab^ |

^a^ statistically significant differences in relation to the control samples, ^b^ statistically significant differences between toxicity of wastewater influent and effluent, ^c^ statistically significant differences between toxicity of wastewater from racks with different frequencies of sewage dosing, ^d^ statistically significant differences between toxicity of control wastewater and sewage containing pharmaceuticals; Mann-Whitney U test, p<0.05

**References**

Pal A, Gin KY, Lin AY, Reinhard M (2010) Impacts of emerging organic contaminants on freshwater resources: review of recent occurrences, sources, fate and effects. Sci Total Environ. https://doi.org/10.1016/j.scitotenv.2010.09.026
